# Whole genome analysis of linezolid resistance in *Streptococcus pneumoniae *reveals resistance and compensatory mutations

**DOI:** 10.1186/1471-2164-12-512

**Published:** 2011-10-17

**Authors:** Dewan S Billal, Jie Feng, Philippe Leprohon, Danielle Légaré, Marc Ouellette

**Affiliations:** 1 Centre de recherche en Infectiologie du Centre de recherche du CHUL and Département de Microbiologie, Infectiologie et Immunologie, Faculté de Médecine, Université Laval, Québec, Canada; 2 State Key Laboratory of Microbial Resources, Institute of Microbiology, Chinese Academy of Sciences, 1 West Beichen Road, Chaoyang District, Beijing 100101, China

## Abstract

**Background:**

Several mutations were present in the genome of *Streptococcus pneumoniae *linezolid-resistant strains but the role of several of these mutations had not been experimentally tested. To analyze the role of these mutations, we reconstituted resistance by serial whole genome transformation of a novel resistant isolate into two strains with sensitive background. We sequenced the parent mutant and two independent transformants exhibiting similar minimum inhibitory concentration to linezolid.

**Results:**

Comparative genomic analyses revealed that transformants acquired G2576T transversions in every gene copy of 23S rRNA and that the number of altered copies correlated with the level of linezolid resistance and cross-resistance to florfenicol and chloramphenicol. One of the transformants also acquired a mutation present in the parent mutant leading to the overexpression of an ABC transporter (spr1021). The acquisition of these mutations conferred a fitness cost however, which was further enhanced by the acquisition of a mutation in a RNA methyltransferase implicated in resistance. Interestingly, the fitness of the transformants could be restored in part by the acquisition of altered copies of the L3 and L16 ribosomal proteins and by mutations leading to the overexpression of the spr1887 ABC transporter that were present in the original linezolid-resistant mutant.

**Conclusions:**

Our results demonstrate the usefulness of whole genome approaches at detecting major determinants of resistance as well as compensatory mutations that alleviate the fitness cost associated with resistance.

## Background

*Streptococcus pneumoniae *is a Gram-positive pathogen responsible for serious diseases such as pneumonia, meningitis, acute otitis media and sepsis. Although vaccination campaigns have been useful at decreasing the prevalence of the most frequent serotypes, the appearance and spread of drug-resistant isolates not included in the initial vaccine formulations are now threatening our capacity at dealing with these infections [1]. The increase in resistance to several classes of antimicrobials due to the clonal spread of few multidrug resistant isolates further worsens the situation. Fortunately, surveillance studies revealed that every strains of *S. pneumoniae *tested were sensitive to linezolid (LNZ) [2-5], the first approved member of the oxazolidinone class of antibiotics. LNZ inhibits the formation of protein synthesis initiation complexes by binding to the central loop segment of domain V of the 23S rRNA [6]. Recent cross-linking and crystallography experiments further revealed that LNZ binds near the catalytic center of the 50S ribosomal subunit and possibly interferes with the placement of the aminoacyl-tRNA [7,8]. LNZ is highly effective against a number of clinically important gram-positive pathogens like *Staphylococcus aureus *and its methicillin-resistant version (MRSA), enterococci and their vancomycin-resistant versions (VRE), and *S. pneumoniae *[9]. Still, *S. pneumoniae *and *Streptococcus oralis *isolates with reduced susceptibility to linezolid (MIC 4 μg/ml) have been already reported [10,11] and the emergence of resistance is unfortunately likely.

Resistance to linezolid in gram-positive bacteria was shown to be associated with key mutations in the domain V of 23S rRNA or in the ribosomal proteins L3 and L4 [12,13]. Although several mutations have been pinpointed, the G2576T transversion in 23S rRNA (when using the *E. coli *numbering system) is the most frequently observed [14] and was shown to occur in clinical isolates of *S. aureus *[13,15], *S. epidermidis *[16-18], *S. hominis *[18], *S. simulans *[18] and in *Enterococci *[19-21] resistant to linezolid. There are four to six gene copies of 23S rRNA in most gram-positive pathogens, and the level of LNZ resistance as well as the fitness cost usually correlates with the number of mutated copies [22-26]. The domain V of 23S rRNA is the binding site of other translation inhibitors like chloramphenicol, florfenicol and quinupristin-dalfopristin, and the G2576T mutation was shown to confer cross-resistance to these antibiotics. A six base pair deletion in the ribosomal protein L4 of *S. pneumoniae *isolates resistant to chloramphenicol was also shown to be associated with non-susceptibility to LNZ [10], and mutations in ribosomal proteins L3 [18,27,28], L4 [18,27,29-31] and L22 [2,18] were further observed in other LNZ-resistant gram-positive bacteria. High levels of resistance to LNZ were shown to be conferred by a plasmid-borne methyltransferase (Cfr) involved in ribosomal protection [32] and recent outbreaks of LNZ-resistant *S. epidermidis *and *S. aureus *have been associated with the clonal spread of Cfr-containing strains [33-35].

Whole genome sequencing of laboratory generated LNZ-resistant *S. pneumoniae *recently revealed mutations in several genes, three of which (a chromosomally-encoded methyltransferase and two ABC transporters) were implicated in resistance to LNZ [29]. Several other mutations were not studied. To improve our understanding of the role of these mutations in LNZ resistance, we used a whole genome transformation approach to reconstruct resistance. DNA extracted from LNZ-resistant mutants was serially transformed in sensitive isolates and the genomes of these transformants were sequenced. We found that the selection of LNZ resistance is accompanied by the acquisition of resistance determinants that confer a fitness cost and of compensatory mutations that partially alleviates the growth defect of the resistant strains.

## Results

The genome sequence of three independent *in vitro*-selected LNZ-resistant *S. pneumoniae *mutants (R6M1, R6M2 and 1974M2) previously revealed the presence of several mutations [29], but the role in resistance had only been studied for some of these. For a more global understanding of the genetic variations associated with resistance to LNZ, we studied a new resistant strain named 1974M1 (LNZ MIC, 32 μg/ml). The transformation of high molecular weight DNA extracted from 1974M1 into *S. pneumoniae *sensitive strains followed by the selection of the transformants under LNZ pressure should allow discriminating the mutations actually involved in resistance from bystander mutations that may have been randomly selected during the selection process. A total of three rounds of transformation of 1974M1 genomic DNA were required to fully reconstruct the high level LNZ resistance of 1974M1 into the *S. pneumoniae *1974 background (leading to the transformant 1974T3) (Table 1, Additional File 1). We also used a similar strategy of transforming the genomic DNA of 1974M1 into the R6 strain but despite several attempts we could not reach a LNZ MIC higher than 16 μg/ml (leading to the transformant R6T2) (Table 1, Additional file 1). Considering the higher initial LNZ MIC of 1974 compared to R6, the fold-increase in resistance was the same between the last two transformants (Table 1).

**Table 1:** Chronological appearance of mutations in *S. pneumoniae *R6 and 1974 transformants at different levels of linezolid resistance^a^.

*S. pneumoniae *strains/transformants^b,c^	LNZ MIC (μg/ml)	No. of colonies tested	spr_ rrnaD^d^	spr_ rrnaC^d^	spr_ rrnaB^d^	spr_ rrnaA^d^	spr0188^e^	spr0196^f^	spr1021^g^
R6	0.5	4	W	W	W	W	W	W	W
R6T1	8	4	M	W	W	M	W	W	W
R6T2	16	4	M	M	M	M	M	M	W
1974	1	4	W	W	W	W	W	W	W
1974T1	8	4	M	M	M	W	W	W	W
1974T2	16	4	M	M	M	W	W	W	W
1974T3	32	4	M	M	M	M	M	M	M

^a ^W (wild-type version); M (1974M1 version).

^b ^R6T1 and R6T2 are first and second level transformants, respectively, of *S. pneumoniae *R6 transformed with genomic DNA extracted from 1974M1.

^c^1974T1, 1974T2 and 1974T3 are first, second and third level transformants, respectively, of *S. pneumoniae *1974 transformed with genomic DNA extracted from 1974M1.

^d ^Mutant 1974M1 had a G2576T mutation in the four copies of spr_rrna23S.

^e ^Mutant 1974M1 had a T409C mutation in spr0188.

^f ^Mutant 1974M1 had a A235G mutation in spr0196.

^g ^Mutant 1974M1 had a G-29T mutation in spr1021 (the number preceded by '-' indicates the position upstream of the ATG).

In order to identify the mutations that had been transferred to 1974T3 and R6T2, the genome sequences of the parent mutant 1974M1 and of both transformants were determined by 454 pyrosequencing. More than 97% of the reads of 1974M1, R6T2 and 1974T3 assembled into 90 to 115 large contigs covering more than 98% of the genome with mean depth coverage of 20X. While we cannot exclude having missed some point mutations, we were capable of fully reconstructing resistance using both a whole genome approach (Table 1) and a targeted approach (see below). Of the fifteen mutations identified in the 1974M1 mutant (Table 2), six mutations were transferred into both 1974T3 and R6T2 transformants (Table 2). These included the G2576T transversion observed in the four copies of the 23S rRNA and missense mutations in the L3 and L16 ribosomal proteins. The only other 1974M1 mutation to be transferred was a G to T transversion that specifically occurred 29 nucleotides upstream of the start codon of spr1021 in the 1974T3 transformant (Table 1). No spontaneous mutations occurred during the selection of the transformant bacteria.

**Table 2:** Common mutations found between the *S. pneumoniae *1974M1 mutant and the transformants resistant to linezolid.

Name/function of genes	Locus Name^a^	1974M1^b,c^	T-1974T3^b,c,d^	T-R6T2^b,e^
23S rDNA	spr_rrnaD23S	G2576T	G2576T	G2576T
23S rDNA	spr_rrnaC23S	G2576T	G2576T	G2576T
23S rDNA	spr_rrnaB23S	G2576T	G2576T	G2576T
23S rDNA	spr_rrnaA23S	G2576T	G2576T	G2576T
50S ribosomal protein L3	spr0188	T409C *Y137H*	T409C *Y137H*	T409C *Y137H*
50S ribosomal protein L16	spr0196	A235G *I79L*	A235G *I79L*	A235G *I79L*
ABC transporter ATP-binding subunit	spr1021	G-29T	G-29T	
Conserved hypothetical/rRNA methyltransferase	spr0333	G503T *S168I*		
Sodium/hydrogen exchanger family protein	spr0573	G141A *T47I*		
Conserved hypothetical protein	spr0855	G25A *A9T*		
Fibronectin-binding protein-like protein A	spr0868	C617A *S206I*		
Conserved hypothetical protein	spr1115	C29T *T10I*		
Transcription antitermination factor	spr1820	G38T *W13L*		
ABC transporter ATP-binding/membrane-spanning protein	spr1885	T490G *S164R*		
ABC transporter ATP-binding/membrane-spanning protein	spr1887	G-32T		

^a ^The *S. pneumoniae *loci number (spr#) are according to the annotation of *S. pneumoniae *R6.

^b ^When the mutations are within coding regions, the change in amino acids is also indicated in italics.

^c ^In noncoding sequences, the number preceded by '-' indicates the position upstream of the ATG.

^d ^DNA of mutant 1974M1 transformed into 1974 (T3 represents third level transformation).

^e ^DNA of mutant 1974M1 transformed into R6 (T2 represents second level transformation).

The targeted sequencing of transformants isolated at different rounds of transformation (R6T1, R6T2 and 1974T1 to 1974T3) revealed that the acquisition of mutations in 23S rRNA began during the first round of transformation in both R6 and 1974 genetic backgrounds while the mutations in the L3 and L16 ribosomal proteins or upstream of spr1021 only occurred during the second and third rounds of transformation (Table 1). The acquisition of mutations in the 23S rRNA is a well established LNZ resistance determinant and the serial transformation of mutated 23S rRNA gene copies also translated into a stepwise increase in LNZ resistance in *S. pneumoniae *R6 in addition to increase cross-resistance to chloramphenicol and florfenicol but not to penicillin (Table 3). We were never able to select for unique integration events of the G2576T mutation in the 1974 background however, as it was always acquired by the four copies of 23S rRNA at the same time (Table 3). Nonetheless, this led to similar fold increase in LNZ resistance than in *S. pneumoniae *R6 (Table 3) and was also linked to chloramphenicol and florfenicol cross-resistance (Table 3). The missense mutations in the 50S ribosomal proteins L3 (spr0188) and L16 (spr0196) of 1974M1 appear to require a specific genetic background for resistance as they were only able to decrease LNZ susceptibility when transformed into a strain in which the four copies of 23S rRNA were mutated (named T-7) (Table 4) and not when transformed into WT strains of *S. pneumoniae *R6 (Table 4) or 1974 (data not shown).

**Table 3:** Relationship between the mutation status at domain V of every 23S rRNA gene and the level of resistance to linezolid and other antibiotics.

*S. pneumoniae *strains	23S rDNA status in PCR fragment/transformant	No. of colonies tested	spr_ rrnaD	spr_ rrnaC	spr_ rrnaB	spr_ rrnaA	MIC LNZ (μg/ml)	MIC CHL (μg/ml)	MIC FFC (μg/ml)	MIC PCG (μg/ml)
R6		4	W	W	W	W	0.5	3	1	0.023
T-R6^23SrDNAR6M2^	G2576T/G2576T	3	M	W	W	W	1	6	2	0.023
T-R6^23SrDNAR6M2^	G2576T/G2576T	4	M	W	W	M	2	12	2	0.023
T-R6^23SrDNAR6M2^	G2576T/G2576T	4	M	M	W	M	4	16	8	0.023
T-R6^23SrDNAR6M2^	G2576T/G2576T	4	M	M	M	M	8	24	16	0.023
1974		4	W	W	W	W	1	3	2	0.023
T-1974^23SrDNA1974M1^	G2576T/G2576T	12	M	M	M	M	16	24	16	0.023

W (wild-type version); M (1974M1 version); LNZ (linezolid); CHL (chloramphenicol); FFC (florfenicol); PCG (penicillin G).

**Table 4:** Functional analysis of mutations in genes spr0188, spr0196, spr0333, spr1021 and spr1887 in resistance to LNZ and other antibiotics in *S. pneumoniae*.

Strains/transformants^a^	Locus^b^	No. of colonies tested	Mutation status 1974M1^c,d^	Mutation status Transformants^c,d^	MIC LNZ^e ^ (μg/ml)	MIC CHL^e ^ (μg/ml)	MIC FFC^e ^ (μg/ml)
R6					0.5	3	2
T-R6^spr0188^	spr0188	8	A409G *Y137H*	A409G *Y137H*	0.5	3	3
T-R6^spr0196^	spr0196	8	T235G *I79L*	T235G *I79L*	0.5	3	2
T-R6^spr1887^	spr1887	8	G-46T	G-46T	0.5	3	2
1974					1	3	2
T-1974^spr0333^	spr0333	8	G626T *G209V*	G626T *G209V*	1	4	4
T-1974^spr1021^	spr1021	3	G-29T	G-29T	2	4	4
							
T-1974^23SrDNA1974M1 ^(T-7)	spr_rrnaDCBA23S		G2576T	G2576T	16	24	16
T-7^spr0333^	spr0333	4	G626T *G209V*	G626T *G209V*	16	24	16
T-7^spr1021^	spr1021	2	G-29T	G-29T	32	32	32
T-7^spr1887^	spr1887	4	G-46T	G-46T	16	24	16
T-7^sp0188^	spr0188	4	A409G *Y137H*	A409G *Y137H*	16	24	16
T-7^sp0188, spr0196^	spr0188		A409G *Y137H*	A409G *Y137H*	32	12	8
	spr0196		T235G *I79L*	T235G *I79L*			

^a ^T stands for transformants; spr0188 (ribosomal protein L3); spr0196 (ribosomal protein L16); spr0333 (conserved hypothetical protein/rRNA methyltransferase) spr1021 (ABC protein-coding gene); spr1887 (*patA *ABC gene).

^b ^The *S. pneumoniae *loci number (spr#) are according to the nomenclature of *S. pneumoniae *R6.

^c ^In noncoding sequences, the number preceded by '-' indicates the position upstream of the ATG.

^d ^When the mutations are within coding regions, the changes in amino acids is indicated in italics.

^e ^LNZ (linezolid); CHL (chloramphenicol); FFC (florfenicol).

**Figure 1:**
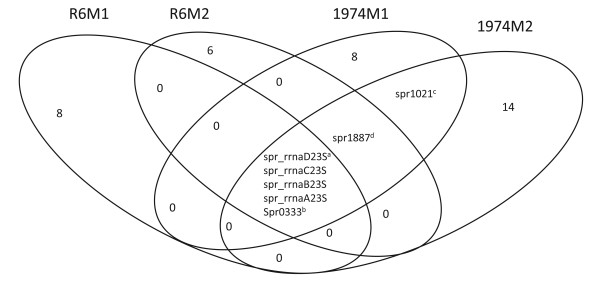
**Venn diagram of the mutations identified in the *S. pneumoniae *R6M1, R6M2, 1974M1 and 1974M2 mutants resistant to LNZ**. ^a ^The four gene copies of 23S rRNA, ^b ^spr0333 codes for a hypothetical protein/rRNA methyltransferase, ^c ^spr1021 codes for the nucleotide-binding subunit of an ABC protein, ^d ^spr1887 codes for an ABC transporter.

The 23S rRNA genes and the rRNA methyltransferase spr0333 were the only mutated genes common to 1974M1 and three other *S. pneumoniae *LNZ-resistant strains described previously [29] (Figure 1). The only other mutations acquired in common by 1974M1 and at least one of these other mutants were a G to T transversion in the promoter regions of the ATP-binding cassette (ABC) genes spr1887 and spr1021 (Table 2, Figure 1). Of these, only the 23S rRNA and spr1021 mutations were transferred to R6T2 and/or 1974T3 (Table 2). When the mutations in or upstream of spr0333, spr1021 or spr1887 were specifically transformed into WT backgrounds of *S. pneumoniae *R6 (data not shown) or *S. pneumoniae *1974 (Table 4), only the mutation upstream of spr1021 was able to increase LNZ resistance and this was correlated with its overexpression (Table 5). Similarly to sensitive isolates, only the mutation linked to spr1021 changed the LNZ susceptibility values when transformed into the T-7 cells (Table 4).

**Table 5:** qRT-PCR to monitor gene expression after introducing a point mutation upstream of spr1021 (G-29T) and spr1887 (G-46T) in the *S. pneumoniae *1974 and T-7 lines.

Strains/transformants	Mean 1974T/1974WT spr1021 expression ratio^a, b, c^	Mean 1974T/1974WT spr1887 expression ratio^a, b, c^
T-1974^spr1021^	3.6 (0.4)	1.2 (0.2)
T-7^spr1021^	3.4 (0.4)	1.2 (0.2)
T-7^spr1021, spr1887^	3.3 (0.5)	12.9 (0.2)
1974	1 (0.2)	1 (0.2)

^a^T (transformant); WT (wild-type)

^b^Values represent an average of three independent experiments.

^c^The standard deviation is indicated in parentheses.

**Figure 2:**
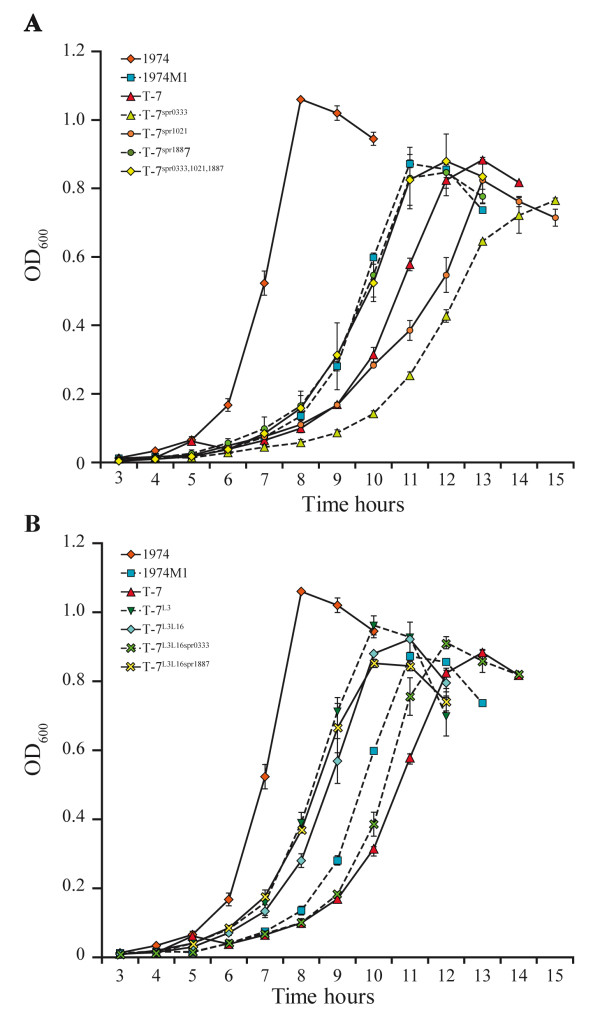
**Growth kinetics of *S. pneumoniae *wild-type (1974), LNZ-resistant mutant (1974M1) and LNZ-resistant transformants**. *A*. Growth curves of *S. pneumoniae *1974 transformed with four mutated copies of 23S rRNA alone (T-7); or along with altered versions of spr0333 (T-7^spr0333^); spr1021 (T-7^spr1021^); spr1887 (T-7^spr1887^); or a combination of the three (T-7^spr0333, 1021, 1887^). *B*. Growth curves of *S. pneumoniae *1974 transformed with four mutated copies of 23S rRNA alone (T-7); or along with altered versions of spr0188 (T-7^L3^); spr0188 and spr0196 (T-7^L3L16^); spr0188, spr0196 and spr0333 (T-7^L3L16spr0333^); or spr0188, spr0196 and spr1887 (T-7^L3L16spr1887^). Data are expressed as the mean of three independent experiments and the statistical significance of the growth differences are indicated in the text.

The acquisition of LNZ resistance conferred a growth defect to the 1974M1 mutant (*p *< 0.01) (Figure 2A, B) and growth kinetic experiments revealed that this was due to several mutations associated with resistance to LNZ. Indeed, the growth of the T-7 transformant harboring four altered copies of 23S rRNA was retarded compared to its 1974 parent (*p *< 0.01), even when compared to the 1974M1 mutant (*p *< 0.01) (Figure 2A). This growth defect was further enhanced by the mutation located within the rRNA methytransferase spr0333 (*p *< 0.01) and by the mutation leading to the overexpression of the ABC gene spr1021 (Figure 2A). The fitness cost conferred by these last two mutations required a background of altered 23S rRNA however, as their transformation failed to affect the growth of *S. pneumoniae *1974 WT (data not shown). In contrast, we found that most 1974M1 mutations that could not be directly linked to LNZ resistance by transformation experiments or the mutations that failed to be transferred to the R6T2 and 1974T3 transformants acted as compensatory mutations involved in increasing the growth fitness of the 1974M1 mutant. Notably, the mutations in the genes coding for the ribosomal protein L3 (Figure 2B) and the mutation leading to the overexpression of the ABC gene spr1887 (Table 5 and Figure 2A) were able to compensate in part for the fitness cost associated with altered copies of 23S rRNA (*p *< 0.01). The mutation upstream of spr1887 was further able to compensate the growth defect conferred by the mutation of the RNA methyltransferase spr0333 and by the overexpression of the ABC gene spr1021 (*p *< 0.01) (Figure 2A). Mutations can thus be involved in either resistance, fitness compensation, or both.

## Discussion

LNZ is a member of the oxazolidinone class of antibiotics that inhibit translation initiation by targeting the domain V of the 23S rRNA. Although resistance to LNZ in clinical settings is rare, and so far absent in the case of *S. pneumoniae *[3,5], the analysis of LNZ-resistant strains revealed the 23S rRNA G2576T mutation to be a major resistance determinant. Other mutations can still be implicated in resistance however, as described in LNZ-resistant *Enterococci*, *Staphylococci *and *S. pneumoniae *[21,22,26,29,36]. Recent advances in DNA sequencing technology make possible the study at the whole-genome level of the genetic bases implicated in the mode of action and resistance mechanisms of drugs [37-42]. To pinpoint the mutations most relevant to LNZ resistance, we sequenced a new *S. pneumoniae *strain, 1974M1, and two *S. pneumoniae *LNZ-resistant transformants generated by whole genome serial transformation of LNZ-sensitive strains with genomic DNA isolated from highly LNZ-resistant mutants.

We first concentrated on recurrent mutations transferred to both transformants as we hypothesized that these would be the most likely to be responsible for resistance. As reported in other bacterial species [22,26,27,29], the G2576T 23S rRNA mutation is also key for LNZ resistance in *S. pneumoniae*. Similarly to *Staphylococcus *and *Enterococci *[12,21,22,26,36], the level of resistance to LNZ in *S. pneumoniae *R6 correlates with the number of mutated gene copies of 23S rRNA. The same gene dosage effect was also observed for the level of cross-resistance to chloramphenicol and florfenicol, two other translation inhibitors acting at the level of domain V of 23S rRNA [43]. In *S. pneumoniae *1974, the four mutated copies were always simultaneously acquired during the targeted transformation of 23S rRNA PCR fragments (12 colonies analyzed). Whether this is due to strain-specific features remains to be established.

The acquisition of 23S rRNA G2576T mutations conferred a biological cost to the *S. pneumoniae *1974M1 mutant (Figure 2). Reduced fitness is a frequent outcome associated with antibiotic resistance [44] and the acquisition of mutations in the primary targets of fluoroquinolones (*gyrA*) [45,46] and β-lactams (penicillin-binding proteins) [47,48] were also shown to confer a growth defect in *S. pneumoniae *resistant mutants. Compensatory mutations occurring either within the genes responsible for resistance or at distinct sites were shown to increase the fitness of the resistant strains, and can even help in achieving higher levels of resistance [49]. Mutations in 50S ribosomal proteins have been observed in LNZ-resistant strains of *S. pneumoniae *and *S. aureus *[10,27,28,50-52] and the selection of a Y137H mutation in the L3 ribosomal protein (spr0188) of 1974M1 is consistent with the reduced LNZ susceptibility previously reported to be conferred by the F147L mutation in ribosomal protein L3 of *S. epidermidis *(which corresponds to amino acid 137 in *S. pneumoniae*) [51]. This mutation failed to directly translate into enhanced levels of LNZ resistance when transferred into R6 WT cells (Table 4) however, although it seems to be implicated in resistance when L16 is mutated and in a context where 23S rRNA is mutated at position G2576T (Table 4). In addition, these mutations are also able to compensate for the growth defect conferred by the 23S rRNA G2576T mutation (Figure 2). Crystallographic studies of the 50S ribosomal subunit have shown that several ribosomal proteins contain extensions approaching 23S rRNA bases near the peptidyltransferase center (PTC), and a critical subset of these proteins includes the L3 and L16 proteins [53,54]. Although they are not part of the PTC *per se*, mutations at residues close to the PTC in L3 and L16 ribosomal proteins could still be implicated in releasing constraints associated with the acquisition of the unfavorable G2576T 23S rRNA mutation by altering the conformation and/or stability of the PTC through changes in second- and third-shell interactions. This could explain the frequent acquisition of ribosomal protein mutations following selection of resistance to LNZ in different bacterial species [3]. The L3 and L16 mutation together conferred increased sensitivity to chloramphenicol and florfenicol however (Table 4), which could be explained by distinct 23S rRNA binding sites compared to LNZ [55].

The selection of a single nucleotide mutation leading to the overexpression of the ABC protein spr1887 (Table 5) in *S. pneumoniae *LNZ-resistant mutants was shown to confer a small but significant increase in the level of resistance to LNZ [29]. Interestingly, we showed here that the increased expression of spr1887 was also able to compensate for the fitness cost conferred by a number of LNZ resistance determinants, including major resistance mechanisms like mutations in 23S rRNAs and more specific ones like mutations in the rRNA methyltransferase spr0333 (Figure 2). The genome of *S. pneumoniae *encodes several ABC proteins, some of which were shown to be involved in drug resistance [56-58]. As mutations at the primary target site (23S rRNA) may lead to excessive concentrations of free LNZ, the cell might require to increase the expression of ABC efflux systems like spr1887 [56,57] to expel the excess of LNZ. Indeed, the decay of antibiotics was shown to generate degradation products displaying potential biological activities and to be detrimental to the strains having acquired primary resistance determinants [59].

## Conclusions

The parent mutants had more mutations than the transformants (Table 1), which suggests that long term step-by-step selection and growth may require additional mutations or that spurious neutral changes are occurring during the selection of resistance. The mutations identified here fully account for the level of LNZ resistance of the mutant and some were found to compensate for a fitness cost. The combination of whole genome transformation and sequencing used here was useful for highlighting mutations playing a dual role in LNZ resistance and fitness compensation.

## Methods

### Bacterial strains, growth conditions and MIC determinations

The R6M1, R6M2, 1974M1 and 1974M2 LNZ-resistant mutants have respectively been generated from the avirulent *S. pneumoniae *strain R6 and the serotype 14 *S. pneumoniae *clinical isolates CCRI-1974 as described previously [29]. The genome sequence of these strains was available which facilitated our approach of whole genome transformation and sequencing. Transformants are described in Tables 1, 2, 3 and 4. Pneumococci were grown in brain heart infusion broth (BHI, Difco) supplemented with 0.5% yeast extract, or in blood agar containing 5% defibrinated sheep's blood. Cultures were incubated for 16-24 hours in a 5% CO_2 _atmosphere at 37°C. The minimal inhibitory concentration (MIC) of drugs was determined by E-test (AB Biodisk) or microdilution. The microdilution assays were performed according to the guidelines of the Clinical and Laboratory Standards Institute (CLSI). The MIC was recorded as the lowest dilution showing no growth. All MIC measurements were done at least in triplicate.

### High molecular weight DNA transformation

High molecular weight genomic DNA was extracted from the LNZ-resistant 1974M1 mutant using the Wizard Genomic DNA Purification Kit (Promega) according to the manufacturer's instructions. All pneumococci strains were made competent as follows. Bacteria were cultured at 37°C in C+Y medium pH6.8 [60] until an optical density at 600 nm (OD_600_) of 0.12. The cells were then concentrated tenfold and resuspended in C+Y media (pH 7.9) with 10% glycerol and frozen at -80°C in 100 μl aliquots. For transformation experiments, the cells were thawed on ice and resuspended in 9 volumes of C+Y media (pH 7.9). The cells were stimulated with 200 ng/ml of competence stimulating peptide-1 at 37°C for 10 minutes. The stimulated competent cells were exposed to approximately 2 μg/ml genomic DNA and incubated at 30°C for 1 hour, followed by 2 hours at 37°C. One-hundred microliters of stimulated cells were then plated on CAT agar supplemented with 5% sheep blood and the appropriate concentration of LNZ.

### Whole-genome sequencing

Genomic DNAs were prepared from mid-log phase cultures of *S. pneumoniae *strains using the Wizard Genomic DNA Purification Kit (Promega) according to the manufacturer's instructions. The genomes of the 1974M1 mutant and the 1974T3 and R6T2 transformants were sequenced using the massively parallel sequencing 454 Life Sciences GS-FLX systems (Roche). Genome sequencing, assemblies and comparative analyses were performed at the McGill University Genome Quebec Innovation Center (http://gqinnovationcenter.com/index.aspx). The R6T2 and 1974T3 sequences produced an assembly of 22× and 23× coverage and an aggregate genome size of 2025687 bp and 1995497 bp, respectively. Whenever possible, the order and orientation of assembled contigs was done in accordance with the genome assembly of *S. pneumoniae *R6 (accession number NC_003098). Mutations deduced from massively parallel sequencing were confirmed by PCR amplification and conventional DNA sequencing. The sequencing data has been deposited at the NCBI under the accession [BioProject: 73475].

### RNA isolation and qRT-PCR

Total RNA was isolated from bacterial cells grown to mid-log phase in BHI using the Qiagen RNeasy Mini Kit (Qiagen) according to the manufacturer's instructions. Genomic DNA contamination was shunned by digesting samples with DNase I (Ambion). The quality and integrity of the starting RNA material were assessed with a 2100 BioAnalyzer and RNA 6000 Nano chips (Agilent). The quality of the RNA was further determined by amplification of housekeeping gene. The cDNAs were generated from total RNAs using the Superscript II reverse transcriptase (Invitrogen) and random hexamers according to the manufacturer's instruction. Real-time quantitative RT-PCR assays were carried out in a BioRad Cycler using SYBR Green I (Molecular Probes). The reactions were carried out in a final volume of 20 μl containing specific primers and iQ SYBR Green Supermix (Bio-Rad). All real-time qRT-PCR data were normalized according to the amplification signals of the 16S rRNA.

### Growth curves and fitness cost determination

The turbidity of *S. pneumoniae *strains grown on blood agar plates was adjusted to 0.5 McFarland units. For each strain, a 1 ml aliquot of 0.5McFarland suspension was inoculated into 99 ml of BHI broth and incubated at 37°C under a 5% CO_2 _atmosphere. Bacterial growth was monitored by recording the OD_600 _at intervals of 1 hour for a total period of 16 hours. Differences in growth rates were measured by Analysis of Variance for statistical significance using the prism software.

## List of abbreviations

bp: base pair; LNZ: linezolid; MIC: minimum inhibitory concentration; rRNA: ribosomal ribonucleic acid; WT: wild-type

## Competing interests

The authors declare that they have no competing interests.

## Authors' contributions

DSB, JF, DL and MO designed the study. DSB performed the experiments and drafted the manuscript. PL helped revise the manuscript and provided critical comments. All authors approved the final version of the manuscript for publication.

## Supplementary Material

Additional file 1 **Whole genome transformation and resistance reconstruction in *Streptococcus pneumoniae***. Figure S1 is a figure describing the strategy to reconstruct resistance to LNZ by using serial whole genome transformation in *S. pneumoniae *R6 and 1974. Click here for file
