# Supplementary material for: Whole genome analysis of linezolid resistance in Streptococcus pneumoniae reveals resistance and compensatory mutations
Source: BMC Genomics. 2011 Oct 17;12:512. doi: 10.1186/1471-2164-12-512 (PMC3212830; doi:10.1186/1471-2164-12-512)
Supplement: Additional file 1 — Whole genome transformation and resistance reconstruction in Streptococcus pneumoniae. Figure S1 is a figure describing the strategy to reconstruct resistance to LNZ by using serial whole genome transformation in S. pneumoniae R6 and 1974. [file 1471-2164-12-512-S1.DOC]

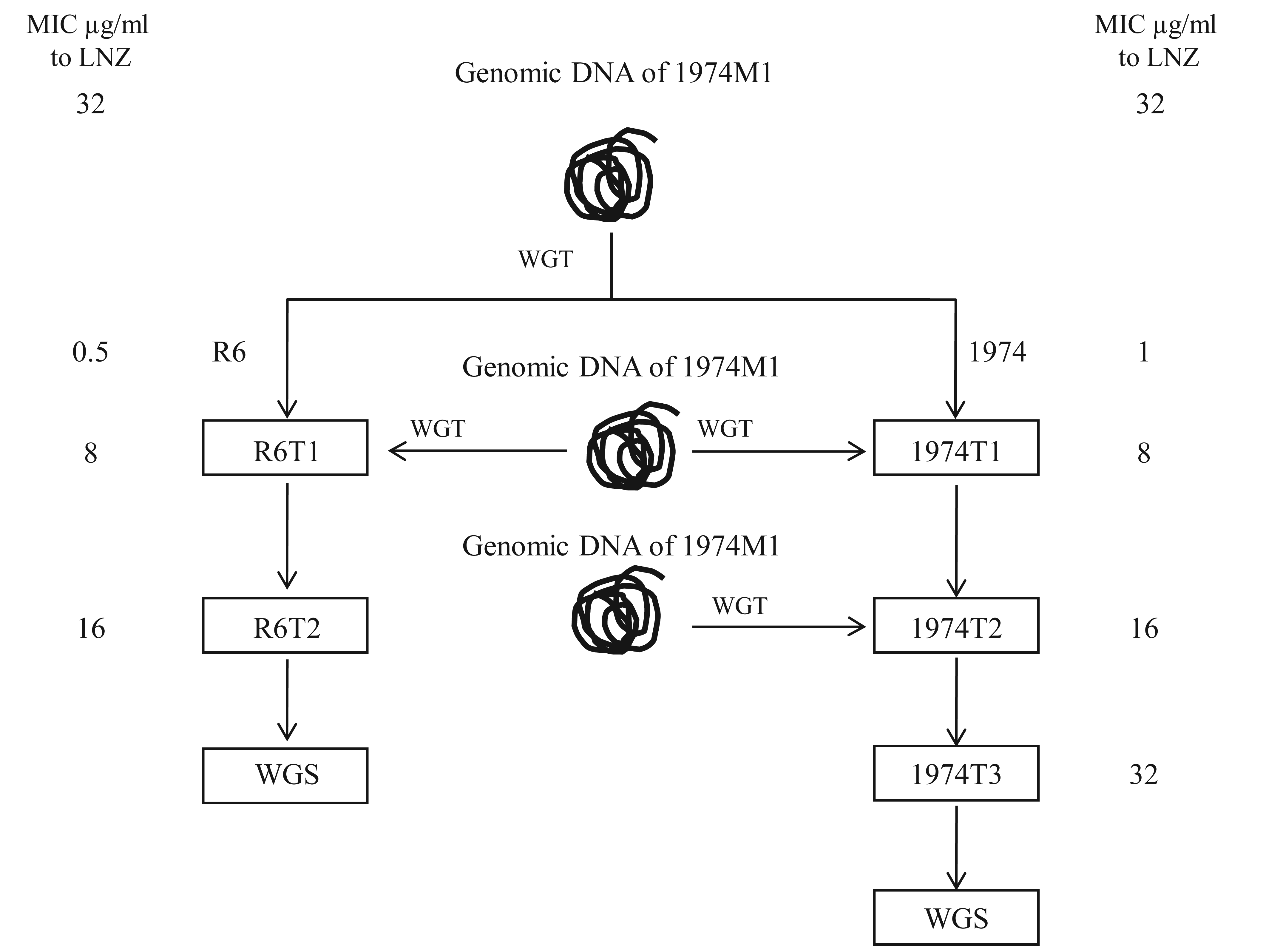


**Figure S1. Whole genome transformation and resistance reconstruction in *Streptococcus pneumoniae.*** Genomic DNA extracted from the *S. pneumoniae* 1974M1 LNZ-resistant mutant was used for the serial transformation of *S. pneumoniae* R6 and 1974 wild-type cells. T1, T2, T3 stand for first, second and third level transformation respectively. WGT (Whole genome transformation), WGS (Whole genome sequencing).
